# Supplementary material for: Genetic rescue increases fitness and aids rapid recovery of an endangered marsupial population
Source: Nat Commun. 2017 Oct 20;8:1071. doi: 10.1038/s41467-017-01182-3 (PMC5715156; doi:10.1038/s41467-017-01182-3)
Supplement: Supplementary file 1 — Supplementary Information [file 41467_2017_1182_MOESM1_ESM.pdf]

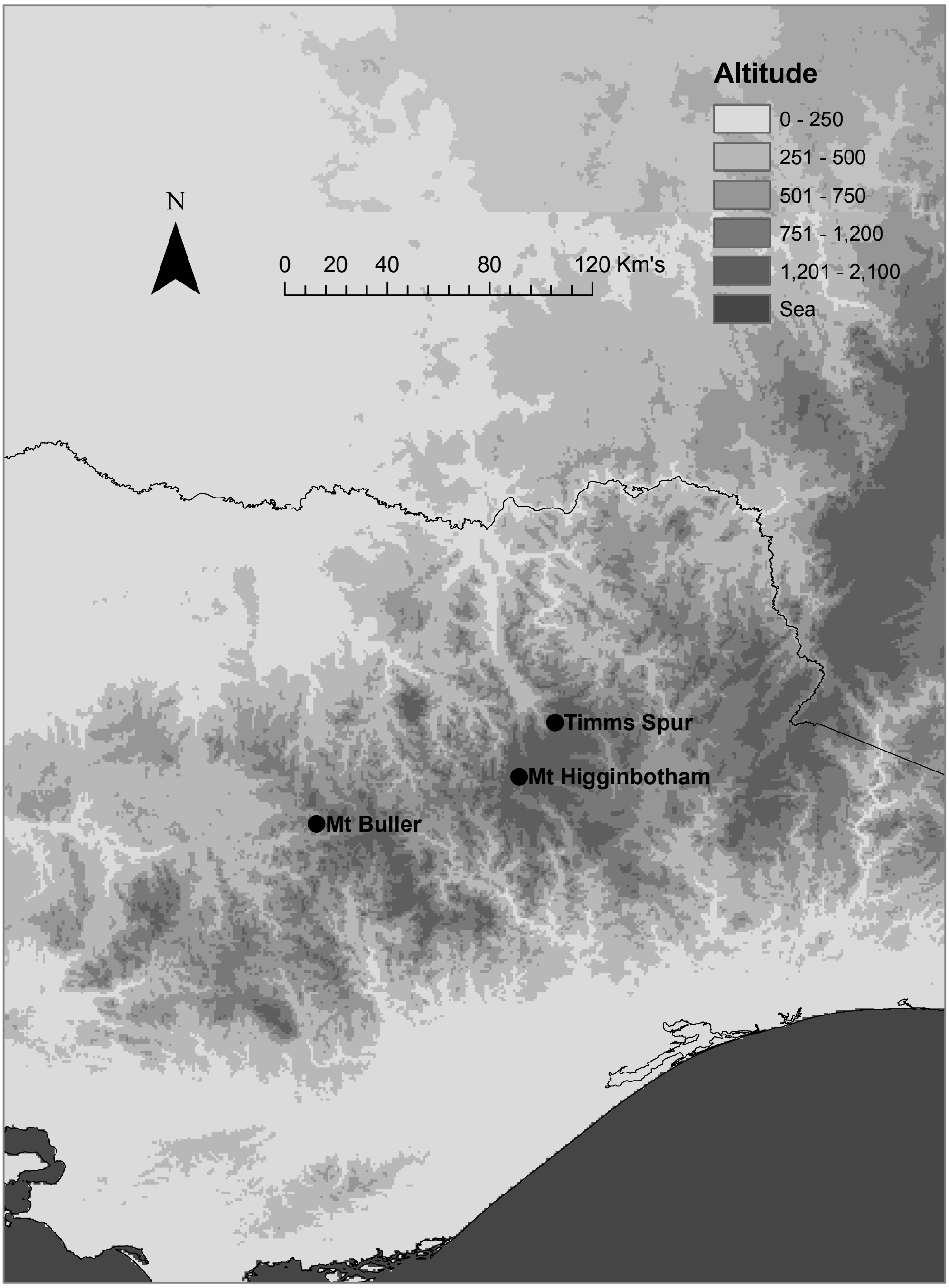

Supplementary Figure 1. Location of Mount Buller, Mount Higginbotham and Timms Spur in Victoria, Australia. Black thin line indicates the state border between Victoria and New South Wales. Altitude is in meters. Source of map data layers: GeoScience Australia (www.ga.gov.au), licensed under the Creative Commons Attribution 4.0 International Licence.

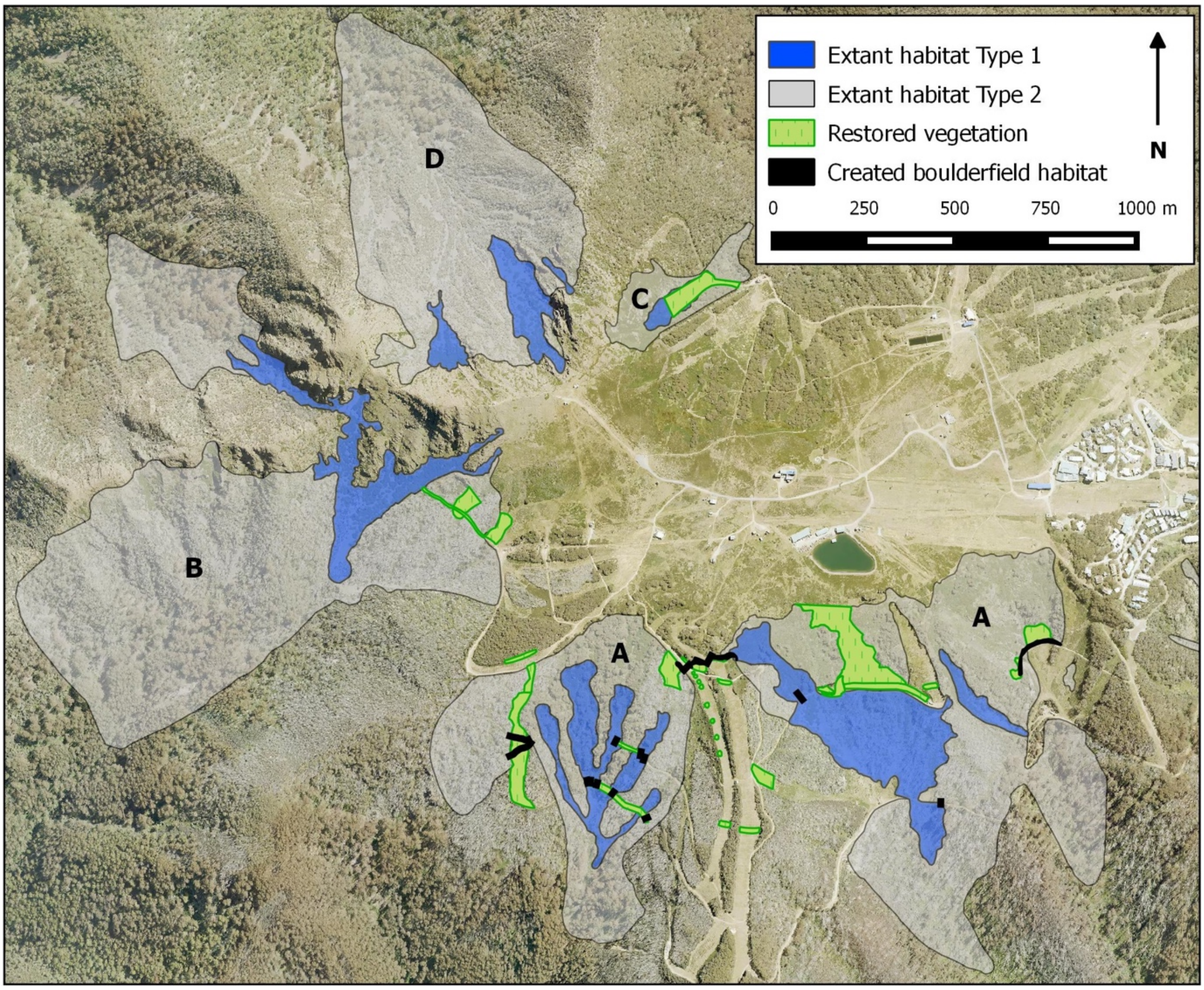

Supplementary Figure 2. Distribution and habitat of *Burramys parvus* at Mount Buller. Region A is the Federation-Wombat bowl, region B south summit, region C Grimmus, and region D north summit. Created boulderfield amounts to approximately 2% of total Type 1 habitat in region A, while restored vegetation amounts to approximately 8.7% of total Type 2 habitat in region A. Source of aerial photo: Mount Buller & Mount Stirling Resort Management, Victoria, Australia.

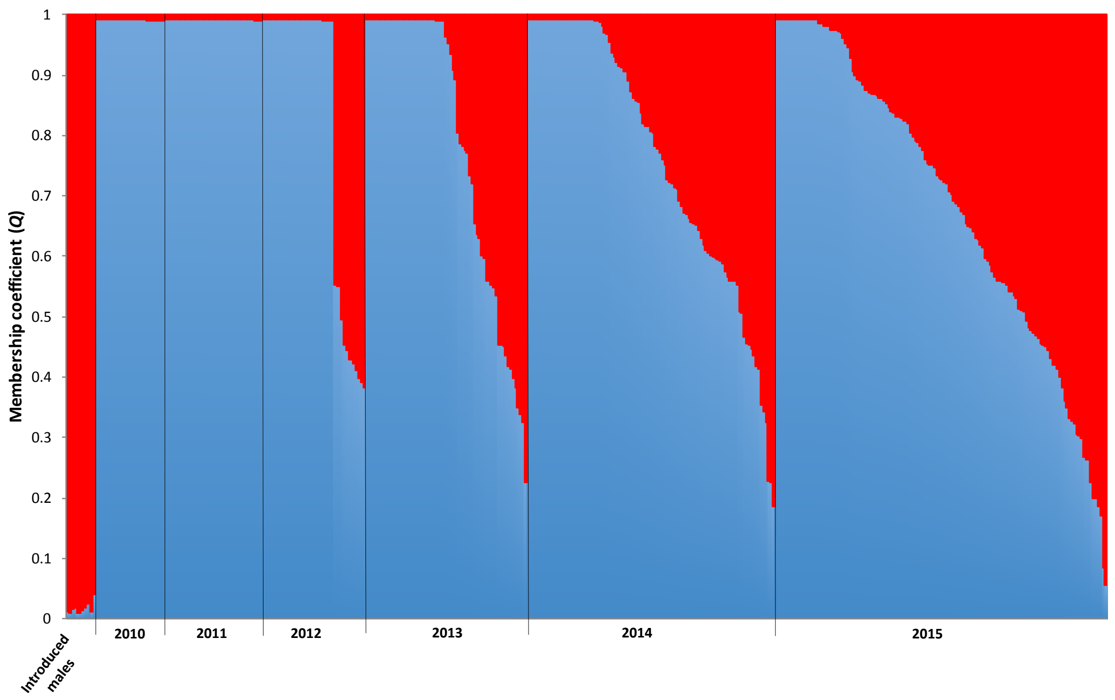

Supplementary Figure 3. Introduced alleles within individuals from the Mt Buller population through time. Estimated membership coefficient (*y* axis) bar plot for individuals (*x* axis) in the Mt Buller population. Each individual is represented by a single vertical bar broken into two coloured segments, where each segment is proportional to the membership coefficient for each of the two (Mount Buller as blue, central region as red) genetic backgrounds. Introduced males/alleles (red) come from two large and healthy populations within the central region (Mount Higginbotham and Timms Spur).

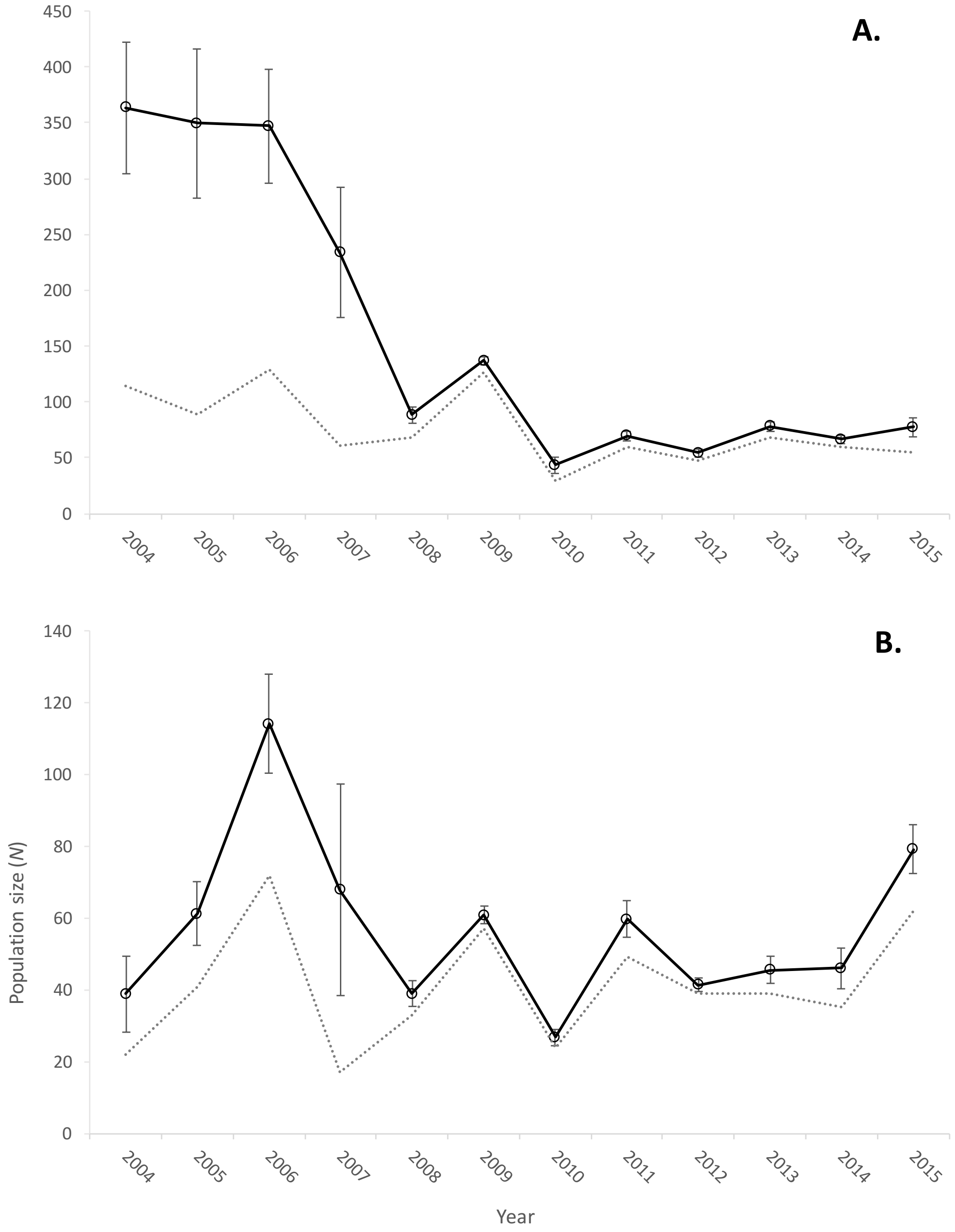

Supplementary Figure 4. *Burramys parvus* adult population size at (A) Mount Higginbotham and (B) Mount Little Higginbotham. Estimates are based on annual spring capture-recapture data for the period 2003-2015 (estimates start from 2004). Solid line is the estimate based on the robust design model with standard error (mean) bars. Dashed line represents the number of unique observed individuals. See Supplementary Methods for exact models used to generate population size estimates.

Supplementary Table 1. Bayesian information criterion (BIC) for candidate models fitted in *RMark*.

Model Number	Model	BIC	No. Par.
1	$s(.)\pi_j(t)p(t+sex)$	9156.84	40
2	$s(.)\gamma'(.)=\gamma"(.)\pi_j(t)p(t+sex)$	9070.83	41
3	$s(sex)\pi_j(t)p(t+sex)$	9133.90	41
4	$s(sex)\gamma'(.)=\gamma"(.)\pi_j(t)p(t+sex)$	9057.37	42
5	$s(sex+t)\pi_j(t)p(t+sex)$	9209.08	58
6	$s(sex+t)\gamma'(.)=\gamma"(.)\pi_j(t)p(t+sex)$	9115.66	59
7	$s(.)\gamma'(.)\gamma"(.)\pi_j(t)p(t+sex)$	8959.57	42
**8**	$\boldsymbol{s(sex)\gamma'(.)\gamma"(.)\pi_j(t)p(t+sex)}$	**8951.29**	**43**
9	$s(sex+t)\gamma'(.)\gamma"(.)\pi_j(t)p(t+sex)$	9018.95	60
10	$s(.)\gamma'(t)\gamma"(t)\pi_j(t)p(t+sex)$	9011.94	75
11	$s(sex)\gamma'(t)\gamma"(t)\pi_j(t)p(t+sex)$	8985.89	76
12	$s(sex+t)\gamma'(t)\gamma"(t)\pi_j(t)p(t+sex)$	9029.83	93
13	$s(t)\gamma'(t)\gamma"(t)\pi_j(t)p(t+sex)$	9016.99	92

Note: Here, BIC is the Bayesian information criterion value and No. Par. is the number of model parameters. The robust design model parameter descriptions are as follows: s is the apparent survival parameter between primary occasions, γ' and γ'' are temporary emigration parameters, π_j for $j = 1,2$ are mixture probabilities and p is the capture probability. $(.)$ indicates the model parameter is constant across primary occasions and (t) indicates the model parameter is time varying. We also use (sex) to indicate that the parameter depends on the sex covariate. There are two types of temporary emigration that can be modelled when using the robust design model, these are: the random type (*i.e.,* temporary emigration occurs randomly) or Markovian type (*i.e.,* an animal 'remembers' that it is off the study area from one occasion to the next). These two

parameters allow us to set the assumed temporary emigration type, such that: when setting $\gamma' = \gamma"$, the temporary emigration is assumed to be random, and when setting γ' and $\gamma"$ as separate parameters the temporary emigration is assumed to be Markovian. If γ' and $\gamma"$ are not included in the models above, then the model does not consider any temporary emigration. The smallest BIC was reported for Model 8, this is highlighted in bold font.

Supplementary Methods

The following gives details on the *R*-scripts, model selection and results used for the capture-recapture analyses on the Mount Buller population (1996-2015), Mount Higginbotham population (2003-2016) and the Mt Little Higginbotham population (2003-2016).

Mount Buller

The dataset is described in detail in Population size estimates in Methods. We fit robust design capture-recapture models using data collected at Mount Buller from years 1996-2015. We model heterogeneity amongst individuals through a mixture-Huggins model and a sex covariate. Model parameters (survival, temporary emigration, and capture probabilities) can be time-varying (i.e., dependent on the capture occasion) or constant. We also allow survival probabilities to be dependent on sex. We fit a set of candidate models using the *RMark R*-package, and use BIC for model selection. Below are the *R*-scripts and outputs.

Load R-script containing the fitting functions and load R-packages:

```
source("RD_functions.r")
library(RMark)
library(ggplot2)
```

Load the data and create data in a readable **RMark** format.

```
## Capture histories.

cap.hist<-as.matrix(read.table("BIG_buller_robust_2015.txt"))

## Vector containing the sex of each individual.

gen.hist<-as.matrix(read.table("BIG_buller_robust_2015_gen.txt"))

# Number of observed individuals in each year.

res_MARK<-as.matrix(read.csv("obs_2015.csv"),header=T)

## Create data in the required RMark format.

collapse.mat<-function(a){paste(a,collapse="")}
rmark.data<-matrix(cbind(apply(cap.hist,1,collapse.mat),as.numeric(rep(1,nrow(cap.hist))),as.numeric(g
en.hist)),nrow(cap.hist),3)
colnames(rmark.data)<-c("ch","freq","sex")
rmark.data<-data.frame(rmark.data)
write.table(rmark.data,"rmark")
data.RMark<-import.chdata("rmark")

data.RMark[,(ncol(data.RMark)-1)]<-as.numeric(data.RMark[,(ncol(data.RMark)-1)])
data.RMark[,ncol(data.RMark)]<-as.numeric(data.RMark[,ncol(data.RMark)])
```

Create a vector of primary capture occasions in the required `RMark` format.

```
cap.occs<-c(20,17,12,8,7,5,8,8,4,5,6,10,8,6,5,9,8,8,9)
```

```
time.intervals<-c()

for(i in 1:(length(cap.occs)))
  {
  a.hist<-c(rep(0,cap.occs[i]-1),1)
  time.intervals<-c(time.intervals,a.hist)
  }

time.intervals<-time.intervals[-length(time.intervals)]
```

Fit models using the `run.robust()` function.

This function takes no input arguments. Comp. times will vary but it usually takes up to 25-30 mins.

```
robust.results<-run.robust()

## No temporary emigration
## model 1: S(~1)Gamma''(~1)Gamma'(~1)pi(~session)p(~session + sex)
## -------------------------------------------------------------
## model 2: S(~1)Gamma''(~1)Gamma'()pi(~session)p(~session + sex)
## -------------------------------------------------------------
## No temporary emigration
## model 3: S(~sex)Gamma''(~1)Gamma'(~1)pi(~session)p(~session + sex)
## -------------------------------------------------------------
## model 4: S(~sex)Gamma''(~1)Gamma'()pi(~session)p(~session + sex)
## -------------------------------------------------------------
## No temporary emigration
## model 5: S(~sex + time)Gamma''(~1)Gamma'(~1)pi(~session)p(~session + sex)
## -------------------------------------------------------------
## model 6: S(~sex + time)Gamma''(~1)Gamma'()pi(~session)p(~session + sex)
## -------------------------------------------------------------
## model 7: S(~1)Gamma''(~1)Gamma'(~1)pi(~session)p(~session + sex)
## -------------------------------------------------------------
## model 8: S(~sex)Gamma''(~1)Gamma'(~1)pi(~session)p(~session + sex)
## -------------------------------------------------------------
## model 9: S(~sex)Gamma''(~1)Gamma'(~1)pi(~session)p(~session + sex)
## -------------------------------------------------------------
## model 10: S(~1)Gamma''(~time)Gamma'(~time)pi(~session)p(~session + sex)
## -------------------------------------------------------------
## model 11: S(~sex)Gamma''(~time)Gamma'(~time)pi(~session)p(~session + sex)
## -------------------------------------------------------------
## model 12: S(~sex + time)Gamma''(~time)Gamma'(~time)pi(~session)p(~session + sex)
## -------------------------------------------------------------
## model 13: S(~time)Gamma''(~time)Gamma'(~time)pi(~session)p(~session + sex)
```

Obtain the results in a table (i.e., BIC values for each fitted model).

```
robust.results$results

##       model
##  [1,] "S(~1)Gamma''(~1)Gamma'(~1)pi(~session)p(~session + sex)"
##  [2,] "S(~1)Gamma''(~1)Gamma'()pi(~session)p(~session + sex)"
##  [3,] "S(~sex)Gamma''(~1)Gamma'(~1)pi(~session)p(~session + sex)"
##  [4,] "S(~sex)Gamma''(~1)Gamma'()pi(~session)p(~session + sex)"
##  [5,] "S(~sex + time)Gamma''(~1)Gamma'(~1)pi(~session)p(~session + sex)"
##  [6,] "S(~sex + time)Gamma''(~1)Gamma'()pi(~session)p(~session + sex)"
##  [7,] "S(~1)Gamma''(~1)Gamma'(~1)pi(~session)p(~session + sex)"
##  [8,] "S(~sex)Gamma''(~1)Gamma'(~1)pi(~session)p(~session + sex)"
##  [9,] "S(~sex + time)Gamma''(~1)Gamma'(~1)pi(~session)p(~session + sex)"
## [10,] "S(~1)Gamma''(~time)Gamma'(~time)pi(~session)p(~session + sex)"
## [11,] "S(~sex)Gamma''(~time)Gamma'(~time)pi(~session)p(~session + sex)"
## [12,] "S(~sex + time)Gamma''(~time)Gamma'(~time)pi(~session)p(~session + sex)"
## [13,] "S(~time)Gamma''(~time)Gamma'(~time)pi(~session)p(~session + sex)"

##       no. pars BIC
##  [1,] "40"     "9156.84"
##  [2,] "41"     "9070.83"
##  [3,] "41"     "9133.9"
##  [4,] "42"     "9057.37"
##  [5,] "58"     "9209.08"
##  [6,] "59"     "9115.66"
##  [7,] "42"     "8959.57"
##  [8,] "43"     "8951.29"
##  [9,] "60"     "9018.95"
```

```
## [10,] "75"      "9011.94"
## [11,] "76"      "8985.89"
## [12,] "93"      "9029.83"
## [13,] "92"      "9016.99"


cleanup(ask=FALSE)
```

Choose the final ``best" model based on the smallest BIC.

The final chosen model was Model 8.

```
## Get final "best" model.

S.time<-list(formula=~sex)
p.time.session=list(formula=~session+sex,share=TRUE)
pi.session<-list(formula=~session)
GammaPrime.dot=list(formula=~1)
GammaDoublePrime.random=list(formula=~1)

model.2.s<-mark(data=data.RMark,model="RDHFHet",time.intervals=time.intervals,
model.parameters=list(S=S.time,GammaPrime=GammaPrime.dot,GammaDoublePrime=GammaDoublePrime.random,p=p.
time.session,pi=pi.session),threads=2,output=F)

cleanup(ask=FALSE)
```

Print the population size estimates and standard errors from the final fitted model.

```
res<-res_MARK2
colnames(res)<-c("year","pop. size"," S.E.","obs.")

round(res,digits=2)

##       year pop. size  S.E. obs.
##  [1,] 1996     92.31  3.23   85
##  [2,] 1997     61.17  1.62   59
##  [3,] 1998     55.50  3.58   48
##  [4,] 1999     37.95  5.24   28
##  [5,] 2000     24.99  3.33   20
##  [6,] 2001        NA    NA   NA
##  [7,] 2002     33.34  7.01   21
##  [8,] 2003     17.12  3.30   13
##  [9,] 2004     21.57  2.05   19
## [10,] 2005      5.22  2.73    3
## [11,] 2006     18.84  6.19   11
## [12,] 2007      9.00  2.18    7
## [13,] 2008     11.18  2.11    9
## [14,] 2009     25.05  3.84   19
## [15,] 2010     37.49  4.09   30
## [16,] 2011     53.39  2.96   48
## [17,] 2012     51.32  3.29   45
## [18,] 2013     85.10  5.82   69
## [19,] 2014    119.67  5.72  102
## [20,] 2015    154.58  5.20  138
```

Mount Higginbotham and Mount Little Higginbotham

Mount Higginbotham (36° 59’ 13.3” S, 147° 08’ 44.1” E) and Mount Little Higginbotham (36° 59’ 33.9” S, 147° 09’ 36.9” E) are both found within the Mount Hotham Alpine Ski Resort in Victoria, Australia (approximately 1.5 km in distance from each other). An environmental improvement program was initiated in 2004 within the Mount Hotham Resort boundaries, similar to that undertaken at Mount Buller Alpine Resort. Live trapping of *B. parvus* was undertaken annually in spring at Mount Higginbotham and Mount Little Higginbotham from

2003-2015. Live trapping occurred at approximately the same time each year (first two weeks of November), with variation in dates dependent on spring snow melt and approximate timing of matings. Trapping was confined to this period to capture females whey they are carrying pouch young. Standard trapping methods (see Trapping in Methods) were followed and undertaken with Elliott type-A live-capture traps (Elliott Scientific, Upwey, Victoria, Australia). Traps were baited with walnuts, wood wool was placed in traps for bedding material, and plastic bags wrapped around the outside for insulation from inclement weather.

Each annual trapping event at both Mount Higginbotham and Mount Little Higginbotham consisted of setting Elliott traps at the same sites in the same grid. A total of 1000 trap nights were undertaken at Mount Higginbotham and 300 at Mount Little Higginbotham on each annual trapping event. First time *B. parvus* captures were ear tagged (National Band & Tag Company, Newport, Kentucky, USA) with a unique number, measured for several morphometric / meristic traits (weight, tail/head/body length, number of pouch young (female), testes size (males), reproductive condition (females and males)), and had a hair or tissue (2 mm ear biopsy) sample taken (stored in > 95% ethanol). For recaptures, ear tag number was recorded and weight, number of pouch young, testes size and reproductive condition were remeasured.

As above for Mount Buller, we fitted robust design capture-recapture models using data collected at each location for the period 2003-2015. We model heterogeneity amongst individuals through a mixture-Huggins model and a sex covariate. Model parameters (survival, temporary emigration, and capture probabilities) can be time-varying (i.e., dependent on the capture occasion) or constant. We also allow survival probabilities to be dependent on sex. We fit a set of candidate models using the *RMark R*-package, and use BIC for model selection. Below are the *R*-scripts and outputs.

Mount Higginbotham dataset:

Load R-script containing the fitting functions and load R-packages:

```
source("RD_functions.r")
library(RMark)
library(ggplot2)
```

Load the data and create data in a readable `RMark` format.

```
## Capture histories.

cap.hist<-as.matrix(read.table("BIG_HB_robust_2015.txt"))

## Vector containg the sex of each individual.

gen.hist<-as.matrix(read.table("BIG_HB_robust_2015_gen.txt"))

# Number of observed individuals in each year.

res_MARK<-as.matrix(read.csv("HB_obs_2015.csv"),header=T)

## Create data in the required RMark format.

collapse.mat<-function(a){paste(a,collapse="")}
rmark.data<-matrix(cbind(apply(cap.hist,1,collapse.mat),as.numeric(rep(1,n
row(cap.hist))),as.numeric(gen.hist)),nrow(cap.hist),3)
colnames(rmark.data)<-c("ch","freq","sex")
rmark.data<-data.frame(rmark.data)
write.table(rmark.data,"rmark")
data.RMark<-import.chdata("rmark")

data.RMark[,(ncol(data.RMark)-1)]<-as.numeric(data.RMark[,(ncol(data.RMark
)-1)])
data.RMark[,ncol(data.RMark)]<-as.numeric(data.RMark[,ncol(data.RMark)])
```

Create a vector of primary capture occasions in the required `RMark` format.

```
cap.occs<-c(5,5,4,5,5,rep(4,9))

time.intervals<-c()

for(i in 1:(length(cap.occs)))
  {
  a.hist<-c(rep(0,cap.occs[i]-1),1)
  time.intervals<-c(time.intervals,a.hist)
  }

time.intervals<-time.intervals[-length(time.intervals)]
```

Fit models using the `run.robust()` function.

This function takes no input arguments. Comp. times will vary but it usually takes up to 25-30 mins.

```
robust.results<-run.robust()

## No temporary emigration
## model 1: S(~1)Gamma''(~1)Gamma'(~1)pi(~session)p(~session + sex)c()
## -------------------------------------------------------------
## model 2: S(~1)Gamma''(~1)Gamma'()pi(~session)p(~session + sex)c()
## -------------------------------------------------------------
## No temporary emigration
## model 3: S(~sex)Gamma''(~1)Gamma'(~1)pi(~session)p(~session + sex)c()
## -------------------------------------------------------------
```

```
## model 4: S(~sex)Gamma''(~1)Gamma'()pi(~session)p(~session + sex)c()
## ------------------------------------------------------------
## No temporary emigration
## model 5: S(~sex + time)Gamma''(~1)Gamma'(~1)pi(~session)p(~session + sex)c()
## ------------------------------------------------------------
## model 6: S(~sex + time)Gamma''(~1)Gamma'()pi(~session)p(~session + sex)c()
## ------------------------------------------------------------
## model 7: S(~1)Gamma''(~1)Gamma'(~1)pi(~session)p(~session + sex)c()
## ------------------------------------------------------------
## model 8: S(~sex)Gamma''(~1)Gamma'(~1)pi(~session)p(~session + sex)c()
## ------------------------------------------------------------
## model 9: S(~sex)Gamma''(~1)Gamma'(~1)pi(~session)p(~session + sex)c()
## ------------------------------------------------------------
## model 10: S(~1)Gamma''(~time)Gamma'(~time)pi(~session)p(~session + sex)c()
## ------------------------------------------------------------
## model 11: S(~sex)Gamma''(~time)Gamma'(~time)pi(~session)p(~session + sex)c()
## ------------------------------------------------------------
## model 12: S(~sex + time)Gamma''(~time)Gamma'(~time)pi(~session)p(~session + sex
)c()
## ------------------------------------------------------------
## model 13: S(~time)Gamma''(~time)Gamma'(~time)pi(~session)p(~session + sex)c()
## ------------------------------------------------------------
## model 14: S(~time)Gamma''(~time)Gamma'(~time)pi(~session)p(~session:mixture + s
ex)c()
## ------------------------------------------------------------
## full model: S(~sex + time)Gamma''(~time)Gamma'(~time)pi(~session)p(~session:mix
ture + sex)c()
```

Obtain the results in a table (i.e., BIC values for each fitted model).

```
robust.results$results

##       model
##  [1,] "S(~1)Gamma''(~1)Gamma'(~1)pi(~session)p(~session + sex)c()"
##  [2,] "S(~1)Gamma''(~1)Gamma'()pi(~session)p(~session + sex)c()"
##  [3,] "S(~sex)Gamma''(~1)Gamma'(~1)pi(~session)p(~session + sex)c()"
##  [4,] "S(~sex)Gamma''(~1)Gamma'()pi(~session)p(~session + sex)c()"
##  [5,] "S(~sex + time)Gamma''(~1)Gamma'(~1)pi(~session)p(~session + sex)c()"
##  [6,] "S(~sex + time)Gamma''(~1)Gamma'()pi(~session)p(~session + sex)c()"
##  [7,] "S(~1)Gamma''(~1)Gamma'(~1)pi(~session)p(~session + sex)c()"
##  [8,] "S(~sex)Gamma''(~1)Gamma'(~1)pi(~session)p(~session + sex)c()"
##  [9,] "S(~sex + time)Gamma''(~1)Gamma'(~1)pi(~session)p(~session + sex)c()"
## [10,] "S(~1)Gamma''(~time)Gamma'(~time)pi(~session)p(~session + sex)c()"
## [11,] "S(~sex)Gamma''(~time)Gamma'(~time)pi(~session)p(~session + sex)c()"
## [12,] "S(~sex + time)Gamma''(~time)Gamma'(~time)pi(~session)p(~session + sex)c(
)"
## [13,] "S(~time)Gamma''(~time)Gamma'(~time)pi(~session)p(~session + sex)c()"
## [14,] "S(~time)Gamma''(~time)Gamma'(~time)pi(~session)p(~session:mixture + sex)
c()"
## [15,] "S(~sex + time)Gamma''(~time)Gamma'(~time)pi(~session)p(~session:mixture
+ sex)c()"
##       no. pars BIC
##  [1,] "30"     "6353.55"
##  [2,] "31"     "6353.55"
##  [3,] "31"     "6337.04"
##  [4,] "32"     "6337.04"
##  [5,] "43"     "6336.11"
##  [6,] "44"     "6336.11"
##  [7,] "32"     "6368.05"
##  [8,] "33"     "6351.66"
```

```
##  [9,] "45"     "6336.11"
## [10,] "55"     "6345.58"
## [11,] "56"     "6330.15"
## [12,] "68"     "6356.61"
## [13,] "67"     "6367.09"
## [14,] "82"     "6421.21"
## [15,] "83"     "6422.07"

cleanup(ask=FALSE);
```

Choose the final ``best" model based on the smallest BIC.

The final chosen model was model 11.

```
## Get final "best" model.

S.time<-list(formula=~sex)
p.time.session=list(formula=~session+sex,share=TRUE)
pi.session<-list(formula=~session)
GammaPrime.dot=list(formula=~time)
GammaDoublePrime.random=list(formula=~time)
model.2.s<-mark(data=data.RMark,model="RDHFHet",time.intervals=time.interv
als,
model.parameters=list(S=S.time,GammaPrime=GammaPrime.dot,GammaDoublePrime=
GammaDoublePrime.random,p=p.time.session,pi=pi.session),threads=2,output=F
)

cleanup(ask=FALSE)
```

Print the population size estimates and standard errors from the final fitted model.

```
res<-res_MARK2[-1,]
colnames(res)<-c("year","pop. size"," S.E.","obs.")

round(res,digits=2)

##       year pop. size  S.E. obs.
##  [1,] 2004    363.29 59.43  114
##  [2,] 2005    349.62 66.54   89
##  [3,] 2006    346.89 50.90  129
##  [4,] 2007    233.47 58.40   61
##  [5,] 2008     88.50  7.31   68
##  [6,] 2009    136.91  3.90  127
##  [7,] 2010     43.25  7.89   29
##  [8,] 2011     69.48  4.27   60
##  [9,] 2012     54.46  3.78   47
## [10,] 2013     77.93  4.30   68
## [11,] 2014     66.44  3.25   60
## [12,] 2015     77.69  8.40   55
```

Mount Little Higginbotham dataset:

Load R-script containing the fitting functions and load R-packages:

```
source("RD_functions.r")
library(RMark)
library(ggplot2)
```

Load the data and create data in a readable `RMark` format.

```
## Capture histories.

cap.hist<-as.matrix(read.table("BIG_LHB_robust_2015.txt"))

## Vector containg the sex of each individual.

gen.hist<-as.matrix(read.table("BIG_LHB_robust_2015_gen.txt"))

# Number of observed individuals in each year.

res_MARK<-as.matrix(read.csv("LHB_obs_2015.csv"),header=T)

## Create data in the required RMark format.

collapse.mat<-function(a){paste(a,collapse="")}
rmark.data<-matrix(cbind(apply(cap.hist,1,collapse.mat),as.numeric(rep(1,n
row(cap.hist))),as.numeric(gen.hist)),nrow(cap.hist),3)
colnames(rmark.data)<-c("ch","freq","sex")
rmark.data<-data.frame(rmark.data)
write.table(rmark.data,"rmark")
data.RMark<-import.chdata("rmark")

data.RMark[,(ncol(data.RMark)-1)]<-as.numeric(data.RMark[,(ncol(data.RMark
)-1)])
data.RMark[,ncol(data.RMark)]<-as.numeric(data.RMark[,ncol(data.RMark)])
```

Create a vector of primary capture occasions in the required `RMark` format.

```
cap.occs<-c(rep(3,4),2,rep(3,9))

time.intervals<-c()

for(i in 1:(length(cap.occs)))
  {
  a.hist<-c(rep(0,cap.occs[i]-1),1)
  time.intervals<-c(time.intervals,a.hist)
  }

time.intervals<-time.intervals[-length(time.intervals)]
```

Fit models using the `run.robust()` function.

This function takes no input arguments. Comp. times will vary but it usually takes up to 25-30 mins.

```
robust.results<-run.robust()

## No temporary emigration
## model 1: S(~1)Gamma''(~1)Gamma'(~1)pi(~session)p(~session + sex)c()
## -------------------------------------------------------------
## model 2: S(~1)Gamma''(~1)Gamma'()pi(~session)p(~session + sex)c()
## -------------------------------------------------------------
## No temporary emigration
## model 3: S(~sex)Gamma''(~1)Gamma'(~1)pi(~session)p(~session + sex)c()
## -------------------------------------------------------------
```

```
## model 4: S(~sex)Gamma''(~1)Gamma'()pi(~session)p(~session + sex)c()
## --------------------------------------------------------------
## No temporary emigration
## model 5: S(~sex + time)Gamma''(~1)Gamma'(~1)pi(~session)p(~session + sex)c()
## --------------------------------------------------------------
## model 6: S(~sex + time)Gamma''(~1)Gamma'()pi(~session)p(~session + sex)c()
## --------------------------------------------------------------
## model 7: S(~1)Gamma''(~1)Gamma'(~1)pi(~session)p(~session + sex)c()
## --------------------------------------------------------------
## model 8: S(~sex)Gamma''(~1)Gamma'(~1)pi(~session)p(~session + sex)c()
## --------------------------------------------------------------
## model 9: S(~sex)Gamma''(~1)Gamma'(~1)pi(~session)p(~session + sex)c()
## --------------------------------------------------------------
## model 10: S(~1)Gamma''(~time)Gamma'(~time)pi(~session)p(~session + sex)c()
## --------------------------------------------------------------
## model 11: S(~sex)Gamma''(~time)Gamma'(~time)pi(~session)p(~session + sex)c()
## --------------------------------------------------------------
## model 12: S(~sex + time)Gamma''(~time)Gamma'(~time)pi(~session)p(~session + sex
)c()
## --------------------------------------------------------------
## model 13: S(~time)Gamma''(~time)Gamma'(~time)pi(~session)p(~session + sex)c()
## --------------------------------------------------------------
## model 14: S(~time)Gamma''(~time)Gamma'(~time)pi(~session)p(~session:mixture + s
ex)c()
## --------------------------------------------------------------
## full model: S(~sex + time)Gamma''(~time)Gamma'(~time)pi(~session)p(~session:mix
ture + sex)c()
```

Obtain the results in a table (i.e., BIC values for each fitted model).

```
robust.results$results

##       model
##  [1,] "S(~1)Gamma''(~1)Gamma'(~1)pi(~session)p(~session + sex)c()"
##  [2,] "S(~1)Gamma''(~1)Gamma'()pi(~session)p(~session + sex)c()"
##  [3,] "S(~sex)Gamma''(~1)Gamma'(~1)pi(~session)p(~session + sex)c()"
##  [4,] "S(~sex)Gamma''(~1)Gamma'()pi(~session)p(~session + sex)c()"
##  [5,] "S(~sex + time)Gamma''(~1)Gamma'(~1)pi(~session)p(~session + sex)c()"
##  [6,] "S(~sex + time)Gamma''(~1)Gamma'()pi(~session)p(~session + sex)c()"
##  [7,] "S(~1)Gamma''(~1)Gamma'(~1)pi(~session)p(~session + sex)c()"
##  [8,] "S(~sex)Gamma''(~1)Gamma'(~1)pi(~session)p(~session + sex)c()"
##  [9,] "S(~sex + time)Gamma''(~1)Gamma'(~1)pi(~session)p(~session + sex)c()"
## [10,] "S(~1)Gamma''(~time)Gamma'(~time)pi(~session)p(~session + sex)c()"
## [11,] "S(~sex)Gamma''(~time)Gamma'(~time)pi(~session)p(~session + sex)c()"
## [12,] "S(~sex + time)Gamma''(~time)Gamma'(~time)pi(~session)p(~session + sex)c(
)"
## [13,] "S(~time)Gamma''(~time)Gamma'(~time)pi(~session)p(~session + sex)c()"
## [14,] "S(~time)Gamma''(~time)Gamma'(~time)pi(~session)p(~session:mixture + sex)
c()"
## [15,] "S(~sex + time)Gamma''(~time)Gamma'(~time)pi(~session)p(~session:mixture
+ sex)c()"
##       no. pars BIC
##  [1,] "30"     "3192.88"
##  [2,] "31"     "3187"
##  [3,] "31"     "3190.96"
##  [4,] "32"     "3185.94"
##  [5,] "43"     "3225.55"
##  [6,] "44"     "3220.97"
##  [7,] "32"     "3191.08"
##  [8,] "33"     "3190.16"
```

```
##  [9,] "45"     "3224.78"
## [10,] "55"     "3232.88"
## [11,] "56"     "3235.34"
## [12,] "68"     "3278.51"
## [13,] "67"     "3273.78"
## [14,] "82"     "3283.74"
## [15,] "83"     "3291.4"

cleanup(ask=FALSE)
```

Choose the final ``best" model based on the smallest BIC.

The final chosen model was model 4.

```
## Get final "best" model.

S.time<-list(formula=~sex)
p.time.session=list(formula=~session+sex,share=TRUE)
pi.session<-list(formula=~session)
GammaDoublePrime.random=list(formula=~1,share=TRUE)
model.2.s<-mark(data=data.RMark,model="RDHFHet",time.intervals=time.interv
als,
model.parameters=list(S=S.time,GammaDoublePrime=GammaDoublePrime.random,p=
p.time.session,pi=pi.session),threads=2,output=F)

cleanup(ask=FALSE)
```

Print the population size estimates and standard errors (S.E.) from the final fitted model.

```
res<-res_MARK2
colnames(res)<-c("year","pop. size"," S.E.","obs.")

round(res,digits=2)

##       year pop. size  S.E. obs.
##  [1,] 2003     38.83 10.48   22
##  [2,] 2004     61.15  8.92   41
##  [3,] 2005    114.12 13.81   72
##  [4,] 2006     87.90  8.95   64
##  [5,] 2007     67.77 29.42   17
##  [6,] 2008     38.91  3.73   33
##  [7,] 2009     60.86  2.45   57
##  [8,] 2010     26.78  2.30   24
##  [9,] 2011     59.66  5.07   49
## [10,] 2012     41.41  1.90   39
## [11,] 2013     45.55  3.74   39
## [12,] 2014     46.11  5.67   35
## [13,] 2015     79.03  6.79   62
```
